# Risk factors for postoperative hypoxemia in patients undergoing Stanford A aortic dissection surgery

**DOI:** 10.1186/1749-8090-8-118

**Published:** 2013-04-30

**Authors:** Yinghua Wang, Song Xue, Hongsheng Zhu

**Affiliations:** 1 Cardiovascular Surgery Department, RenJi Hospital, School of medicine, Shanghai JiaoTong University, 1630 Dongfang Rd., Shanghai 200127, China

## Abstract

**Background:**

The purpose of this study is to identify the risk factors for postoperative hypoxemia in patients with Stanford A aortic dissection surgery and their relation to clinical outcomes.

**Methods:**

Clinical records of 186 patients with postoperative hypoxemia in Stanford A aortic dissection were analyzed retrospectively. The patients were divided into two groups by postoperative oxygen fraction (PaO2/FiO2):hypoxemia group (N=92) and non-hypoxemia group (N=94).

**Results:**

We found that the incidence of postoperative hypoxemia was 49.5%. Statistical analysis by t-test and χ2 indicated that acute onset of the aortic dissection (p=0.000), preoperative oxygen fraction (PaO2/FiO2) ≤200 mmHg(p=0.000), body mass index (p=0.008), circulatory arrest (CA) time (p=0.000) and transfusion more than 3000 ml(p=0.000) were significantly associated with postoperative hypoxemia. Multiple logistic regression analysis showed that preoperative hypoxemia, CA time and transfusion more than 3000 ml were independently associated with postoperative hypoxemia in Stanford A aortic dissection.

**Conclusion:**

Our results suggest that postoperative hypoxemia is a common complication in patients treated by Stanford A aortic dissection surgery. Preoperative oxygen fraction lower than 200 mmHg, longer CA time and transfusion more than 3000 ml are predictors of postoperative hypoxemia in Stanford A aortic dissection.

## Background

Aortic dissection can be divided into type A and type B by Stanford classification and is one of the most urgent medical cases for its high mortality and morbidity. Although open heart surgery for treating Stanford A aortic dissection has been a universal option, the patients are still threatened by the lethal perioperative complications such as hemorrhage, hypoxemia, renal dysfunction, cerebral dysfunction and etc. Postoperative hypoxemia is defined as an oxygen fraction (PaO_2_/FiO_2_) ≤200 mmHg under mechanical ventilation in the first 24 hour after surgery [1]. As a matter of fact, postoperative hypoxemia resembles ARDS (acute respiratory distress syndrome) when oxygen fraction is below 200 mmHg.

Patients who undergo cardiac surgery generally have a more or less increased risk of postoperative hypoxemia. Postoperative hypoxemia has been reported to occur in 12.2-27.1% of patients after cardiopulmonary bypass, and in as high as 51% after open heart aortic dissection surgery [1-3]. Hypoxemia is a dangerous complication and usually accompanied by a number of untoward consequences such as prolonged ventilator support, longer stay in ICU and hospital. Prolonged mechanical ventilation would lead to more and serious complications. Continuous postoperative hypoxemia may cause dysfunctions of other organs besides lungs. The aim of our study is to evaluate the risk factors of postoperative hypoxemia in Stanford A aortic dissection and its impact on the clinical outcomes.

## Methods

From Dec 2004 to July 2012,186 consecutive patients with acute Stanford A aortic dissection underwent open heart surgery in RenJi Hospital. This study was approved by RenJi Hospital Clinical Research Ethics Committee(2012027) Acute Stanford A aoritic dissection was diagnosed by enhanced computed tomography scan and aoritc valve regurgitation was confirmed by echocardiography. About 70% of the patients underwent surgical procedure within 48 hours, others within 21 days. 4 patients who died during the first 48 hours after surgery were the result of multiorgan dysfunction syndrome and refractory bleeding. They have been excluded from the 186 patients above. In the present study, namely postoperative hypoxemia was defined as a PaO2/FiO2 ≤200 mmHg in the first 24 hour after surgery, according to the diagnostic criteria for ARDS established by American-European consensus conference. The 186 patients were divided into two groups by postoperative oxygen fraction (PaO2/FiO2): hypoxemia group(N=92) and non-hypoxemia group (N=94). We compared 17 perioperative factors between the two groups. Mortality, morbidity and long-term survival were compared between the two groups. In-hospital outcome variables were obtained from patients’ medical records. Follow-up information including survival, cardiovasucular-related events and causes of death were obtained through out-patient clinic visit or from telephone correspondence with the patients or their relatives.

### Surgical procedure

All operations were performed through a median sternotomy. After heparinization, cardiopulmonary bypass was established by cannulation of right atrium or separately the superior and inferior vena cava. The right or left femoral artery or the right subclavian artery was optionally the site of cannulation. A left ventricular vent line was inserted through the right superior pulmonary vein. Once the cardiopulmonary bypass started, systemic cooling was initiated. After clamping the ascending aorta, the root of aorta was opened and cardioplegic solution was infused through direct selective cannulation of the coronary ostia and the intimal tear and entry of the dissection was detected. When the proximal aortic root procedure was finished, the rectal temperature was around 18°C. The circulation was arrested, crossclamping removed and the intimal tear or entry of dissection of aortic arch and proximal descending aorta was explored and identified. If the entry was close to the orifice of the arch vessels, aortic arch was totally replaced, and if the entry was localized in the lesser curvature of the transverse aortic arch proximal to the left subclavian artery, hemiarch replacement or intraoperative descending aorta endograft deployment was performed. When the intimal tear involved the ascending aorta and the entry site could not be identified, the entire ascending aorta was replaced. Aortic root replacement, Bentall procedure would be performed for patients with dilation of aortic root, especially in Marfan’s syndrome patients. Antegrade selective cerebral perfusion was carried out when we expected that the circulatory arrest time would be longer than 30 min or determined by the surgeon’s personal preference and experience. With the help of antegrade selective cerebral perfusion, the rectal temperature may be maintained at about 25°C during circulatory arrest. The operative variables and concomitant procedures are shown in Table 1.Arch-involved procedure was performed in 84 patients, including 48 total arch replacement, 14 hemiarch replacement and 22 intraoperative aortic arch endograft deployment. Concomitant procedures included coronary artery bypass grafting in 4 patients, mitral valve replacement in 4 patients and atrial septal defect repair in another 2 patients.

**Table 1:** Operative variables and concomitant procedures

**Operative data**	**Total (n=186)**
**Arterial cannulation**	
Femoral artery	178
Right subclavical artery	8
**Venous canulation**	
Bicaval	6
Right atrium	180
**Perfusion technique**	
ECC+DHCA	178
ECC+ASCP	8
**Operative procedures**	
Arch-involved procedure	84
Total arch replacement (TAR)	48
Hemiarch replacement (HAR)	14
Intraoperative aortic arch endograft deployment (IAAED)	22
Intraoperative descending aorta endograft deployment (IDAED)	66
Wheat procedure	6
Wheat+ TAR+IDAED	2
Bentall’s procedure	34
Bentall’s +HAR	4
Bentall’s +IAAED	4
Bentall’s +TAR+IDAED	8
Bentall’s +IDAED	2
Ascending aorta replacement (AAR)	40
AAR+IAAED	18
AAR+TAR	4
AAR+HAR	10
AAR+IDAED	20
AAR+TAR+IDAED	34
**Concomitant procedures**	
Cornary artery bypass grafting	4
Mitral valve replacement	4
Atrial septal defect repair	2
CPB time	184.85±36.83
AO time	94.10±34.58
ASCP time	50.63±2.39
CA time	26.19±9.91

ECC:extracorporeal circulation; ASCP:antegrade selective cerebral perfusion; IAAED:Intraoperative aortic arch endograft deployment; IDAED:Intraoperative descending aorta endograft deployment; CPB:cardiopulmonary bypass; AO:aortic clamping; CA:circulatory arrest.

### Statistical analysis

**Figure 1:**
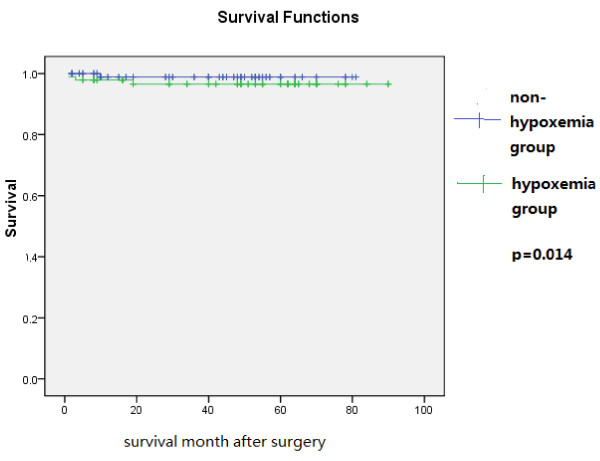
Kaplan-Meier survival curve.

All statistical analysis were performed with SPSS16.0 for windows. Quantitative variables were described as mean and standard deviation. The test of homogeneity variance was used for determination of quantitative data distribution. When the distribution of variables was normal, independent samples t-test was used for comparison of quantitative sizes of two independent samples. The dependence of qualitative variable was evaluated by Chi-squared (χ2) criterion. Multivariate stepwise forward logistic regression analysis was adopted to identify independent risk factors for postoperative hypoxemia. Survival was analyzed by the Kaplan-Meier method and log rank test. A p value of less than 0.05 was considered statistically significant. Kaplan-Meier curves were shown in Figure 1.

## Results

From Dec 2004 to July 2012,186 patients with acute Stanford A aortic dissection underwent open heart surgery in RenJi Hospital. The age of the patients was 51.42±8.93 years, ranging from 28 to 76 years. 70.97% of them were male and 78.5% were diagnosed as acute aortic dissection. The overall in-hospital mortality was 1.1%. The perioperative variables of patients were shown in Table 2.

**Table 2:** Preoperative patient characteristics

**Characeristics**	**Total (n=186)**
**Age (years), M±SD**	51.42±8.93
**Gender (male%)**	70.97
**Height (cm), M±SD**	169.19±8.83
**Weight (kg), M±SD**	70.94±15.23
**BMI (kg/m2), M±SD**	24.68**±**4.48
**History of smoking (%)**	32.8
**Acute onset (%)**	78.49
**Preoperative hypoxemia (%)**	49.46
**History of previous cardiac operation (%)**	4.30
**Preoperative LVEF, M±SD**	55.54**±**7.50
**Hypertension (%)**	57.5
**Diabetes mellitus (%)**	5.38

BMI-body mass index, LVEF-left ventricular ejection fraction.

M±SD--mean±standard deviation.

As shown in Table 2, the incidence of postoperative hypoxemia in our patient population was 49.5%. In order to determine the factors associated with postoperative hypoxemia, 186 patients were divided into two groups by their postoperative oxygen fraction (PaO2/FiO2). Patients with posteroperative PaO2/FiO2 ≤200 mmHg were classified into the hypoxemia group, while others into the non-hypoxemia group. Univariate analyses of perioperative characteristics in these two groups were shown in Table 3. Significant differences between the two groups were found in following clinical variables: acute onset of the aortic dissection (p=0.000), preoperative oxygen fraction (PaO2/FiO2) ≤200 mmHg (p=0.000), body mass index (p=0.008), circulatory arrest (CA) time(p=0.000) and transfusion more than 3000 ml (p=0.000).

**Table 3:** Univariate analysis in postoperative hypoxemia

**Characteristics**	**Hypoxemia group**	**Non-hypoxemia group**	**P value**
Age (years), mean (sd)	51.70 (9.16)	51.15 (8.74)	NS
Gender (male) (%)	50	50	NS
Height (cm), mean (sd)	168.26 (8.48)	170.11 (9.10)	NS
Weight (cm), mean (sd)	72.48 (15.16)	69.44 (15.22)	NS
BMI (kg/m2), mean (sd)	25.55 (4.69)	23.83 (4.12)	P=0.008
History of smoking (%)	57.4	42.6	NS
Acute onset (%)	56.2	43.8	P=0.000
Preoperative hypoxemia (%)	76.1	23.9	P=0.000
History of previous cardiac operation (%)	25	75	NS
Preoperative LVEF (%), mean (sd)	56.50 (5.55)	54.60 (8.94)	NS
Hypertension (%)	53.3	46.7	NS
Diabetes mellitus (%)	80	20	NS
CPB time (min), mean (sd)	185.50 (37.61)	184.21 (36.24)	NS
AO time (min), mean (sd)	94.86 (33.97)	93.35 (35.33)	NS
CA time (min), mean (sd)	29.29 (10.29)	23.17 (8.54)	P=0.000
Postoperative LVEF<40% (%)	66.7	33.3	NS
mechanical ventilation duration (hour), mean (sd)	79.22 (62.31)	41.45 (5.54)	NS
Postoperative transfusion ≥3000 ml (%)	76.1	23.9	P=0.000

BMI-body mass index, LVEF-left ventricular ejection fraction.

CPB-cardiopulmonary bypass AO-aortic clamping.

CA- circulatory arrest.

m(sd)—mean(standard deviation).

As shown in Table 4, multiple logistic regression analysis confirmed that preoperative hypoxemia, CA time and transfusion more than 3000 ml were independently associated with postoperative hypoxemia in Stanford A aortic dissection.

**Table 4:** Multivariate logistic regression analysis model for postoperative hypoxemia

**Variables in the equation**									
		**B**	**S.E.**	**Wald**	**df**	**Sig.**	**Exp(B)**	**95.0% C.I. for EXP(B)**	
								**Lower**	**Upper**
Step 1^a^	BMI	.020	.050	.151	1	.697	1.020	.924	1.125
	Acute onset	-.859	.626	1.886	1	.170	.424	.124	1.444
	Preoperative hypoxemia	2.682	.528	25.758	1	.000	14.611	5.187	41.158
	CA time	.078	.023	11.286	1	.001	1.081	1.033	1.132
	transfusion≥3000 ml	2.256	.425	28.211	1	.000	9.541	4.151	21.932
	Constant	−4.368	1.386	9.924	1	.002	.013		

a. Variable(s) entered on step 1: BMI, acute onset of aortic dissection, preoperative hypoxemia, CA time, transfusion ≥3000 ml.

BMI-body mass index.

CA- circulatory arrest.

All patients have been followed up through out-patient clinic visit or by telephone contacts. The follow-up period was from one month to ninety months (43.65±23.70 month),with a period of 47.96±24.10 month for hypoxemia group and 39.44±22.64 month for non-hypoxemia group. Four patients died, two died of respiratory failure during the first three month after operation in the hospital, others died of stroke or sudden death during follow-up period. Kaplan-Meier survival curve was shown in Figure 1 which revealed a significant difference in the 90-month survival between the hypoxemia group and non-hypoxemia group(p=0.014).

## Discussion

Stanford A aortic dissection has been known for its high mortality and morbidity. Since deep hypothermia, ultrafiltration and selective cerebral perfusion technique were applied in surgery for Stanford A aortic dissection, the incidence of postoperative cerebral complications has decreased. Hypoxemia, as another lift-threatening postoperative complication for Stanford A aortic dissection, may be attributed to prolonged mechanical ventilation and ICU-stay. Acute aortic dissection is frequently accompanied by hypoxemia. Yet currently, few studies revealed the clinical entity in such patients and the management after surgery for acute Stanford A aortic dissection surgery. The aim of our study is to reveal the incidence, risk factors and management of hypoxemia after the surgery for acute Stanford A aortic dissection.

Previous studies disclosed that persistent hypoxemia was the most common cause of PMV (prolonged mechanical ventilation) and ARDS was the most common cause of hypoxemia [4-6]. The etiology of acute respiratory distress syndrome and its less severe form acute lung injury (ALI) may be systemic, rather than pulmonary confirmed to. One of the diagnostic criteria of ARDS is oxygenation impairment (PaO2/FiO2 ≤200 mmHg). Nevertheless, the respiratory failure in patients with hypoxemia in this study could not be diagnosed technically as ARDS or ALI, because it was not usually accompanied by typical radiological pulmonary infiltrates. Therefore, in the present study, postoperative hypoxemia was defined as PaO2/FiO2 ≤200 mmHg in the first 24 hour after surgery, according to the diagnostic criteria for ARDS established by American-European consensus conference [4-8].

Patients who undergo cardiac surgery have an increased risk of postoperative hypoxemia [5]. Postoperative hypoxemia has been reported to occur in 12.2%-27.1% of patients after cardiopulmonary bypass and usually is 51% in open heart aortic dissection surgery [1-3]. In this study, the incidence of postoperative hypoxemia is 49.5% in open heart Stanford A dissection surgery. Previously reported risk factors for hypoxemia after cardiac surgery include advanced age, obesity, smoking history, previous heart surgery, emergency surgery, low LVEF, preoperative chronic pulmonary disease, preoperative myocardial infarction, preoperative diabetes, cardiogenic and noncardiogenic pulmonary edema, pneumonia, excessive blood transfusion and prolonged CPB time [9-11]. In the present study, we identified BMI, preoperative hypoxemia (PaO2/FiO2) ≤200 mmHg, acute onset of Stanford A aortic dissection, CA time, postoperative transfusion ≥3000 ml as the risk factors for postoperative hypoxemia after Stanford A aortic dissection surgery.

Postoperative hypoxemia is a common complication in cardiac surgery. CPB (cardiopulmonary bypass) and CA(circulatory arrest) have been reported to play important roles in the postoperative hypoxemia [12-16]. Stanford A aortic dissection surgery is associated with a high incidence of postoperative hypoxemia compared with other elective cardiac surgical procedures [5,14-16]. During cardiac surgery, both experimental and clinical studies have well documented that open heart surgery with CPB could result in systemic inflammatory response, activation of complements, thrombin, cytokines, endothelin, endotoxins, neutrophils, adhesion molecule, macrophages, multiple inflammatory mediators and impaired immune reaction and organ dysfunction [17-23]. As CPB represents a kind of non-physiological circulation affecting the peripheral tissue perfusion, particularly in the case of prolonged pump time, which might lead to impaired capillary membrane permeability, malperfusion, tissue anoxia and pulmonary complications [24-27]. Although deep hypothermia and antegrade selective cerebral perfusion may preserve better cerebral function during circulatory arrest, the malperfusion of other organs may still lead to generally ischemic reperfusion injury. A prospective study by De Santo et al. analysed the effect of pulmonary perfusion during hypothermic circulatory arrest which may preserve better lung function but that was not used in our study [28].

Deep hypothermia is an important technique commonly used in acute Stanford A aortic dissection surgery. However, deep hypothermia participates in both platelet activation pathways and the enzymatic activity of clotting factors which may lead to bleeding and subsequent transfusion. Excessive transfusion is attributed to active bleeding or coagulation abnormalities. Loss of coagulation factors, destruction of platelets and microthrombosis may all lead to lung injury [29-31]. In our study, prolonged CA time and transfusion more than 3000 ml are associated with the hypoxemia.

According to our experience, large dose of methylperdnisolone in the operation and a rather small dose of methylperdnisolone during the first three day after the operation are regularly used in order to decrease the inflammatory reaction. We also run the operation and control bleeding as fast as possible to reduce the CPB or DHCA (deep hypothermic circulatory arrest) time and excessive transfusion.

In the present study, 78.49% of patients who were sent to emergency department for acute onset of Stanford A aortic dissection were found to be hypoxemia (PaO2/FiO2 ≤200 mmHg) before the operation. In our study, all patients breathed spontaneously with nasal prongs or face mask with inhaled oxygen 5-8 L/min before operation. The arterial blood gas and preoperative PaO2/FiO2 were calculated before machanical ventilation. Both acute onset of dissection and preoperative hypoxemia are associated with postoperative hypoxemia. Acute onset of aortic dissection indicates sudden rupture of intima, propagation of the dissection into the medial layer, acute bleeding and activation of cellular and humoral inflammatory systems. In the process of acute bleeding, ventilation and perfusion are out of balance, which leads to hypoxemia. Quite a number of investigators reported that oxygenation was impaired from the onset of dissection due to the inflammatory cascade reaction, increased endothelial and epithelial permeability, increased pulmonary vascular pressure and impaired alveolar surfactant function, which can lead to decrease in the preoperative PaO2/FiO2 ratio. The preoperative inflammatory status of the patient significantly affects the degree of systemic inflammatory response during the Stanford A aortic dissection surgery in succession [1,9]. Therefore, postoperative hypoxemia may be related to preoperative disturbance in oxygenation. In our opinion, we try all the best to maintain the perioperative fluid balance and meticulous ventilation manipulation, especially in the very first day after the operation.

Obesity have been reported as predictors for hypoxemia [4,32]. Consistently, we found that obese patients, with BMI>25 kg/m2 are more likely to develop postoperative hypoxemia. It is well-known that obesity is frequently accompanied by some co-morbidity, including obstructive sleep apnea and hypoventilation syndrome which all related to hypoxia [32,33]. In the present study, the body mass index showed a significant influence in univariate analysis, but not in the multivariate regression model.

In our study, the long-term survival in non-hypoxemia group was better than that in hypoxemia group, which was in accordance with previous studies [13]. Survivors following cardiac surgery are found to enjoy a good quality of life after discharge.

## Conclusions

In conclusion, our study demonstrated that BMI (Body mass index), preoperative hypoxemia (PaO2/FiO2 ≤200 mmHg),acute onset of Stanford A aortic dissection, CA time, postoperative transfusion ≥3000 ml were the key risk factors for Stanford A aortic dissection surgery. And preoperative hypoxemia, CA time and transfusion ≥3000 ml were independent predictors of postoperative hypoxemia after Stanford A aortic dissection surgery. Optimized treatments including preoperative respiratory tract management to control those predictors should be helpful to improve the clinical outcomes.

### Limitation

Like all retrospective studies, this investigation also has several limitations. First, the patient population collected for investigation was relatively small. Second, owing to the emergency nature of the Stanford A aortic dissection surgery, detailed information about preoperative pulmonary disease was not completely available. The presence of COPD (chronic obstructive pulmonary disease) was judged on the basis of the patient’s medical history which might not be well defined. Finally, as this is a retrospective study, the potential misclassification bias could not be completely excluded.

## Competing interests

The authors declare that they have no competing interests.

## Authors’ contributions

SX performed all the surgeries in our study. HZ helped to design the study and revise the grammar and idiomatic expression. YW participated in design of the study, statistically analysis and draft of the manuscript. All authors read and approved the final manuscript.
